# Targeted vs Standard Contact Precautions and Transmission of Methicillin-Resistant *Staphylococcus aureus* and Vancomycin-Resistant *Enterococci* in Critically Ill Adults: A Pilot Clinical Trial

**DOI:** 10.1016/j.chstcc.2026.100282

**Published:** 2026-05-25

**Authors:** Luis E. Huerta, George E. Nelson, Matthew W. Semler, Thomas R. Talbot, Michael J. Noto, Jonathan P. Wanderer, Robert E. Freundlich, C. Buddy Creech, Isaac P. Thomsen, Nicole Soper, Christopher J. Lindsell, Samuel Nwosu, Todd W. Rice

**Affiliations:** Division of Pulmonary, Critical Care, and Sleep Medicine, Department of Medicine, Keck School of Medicine of USC, Los Angeles, CA; Division of Infectious Diseases, Vanderbilt University Medical Center, Nashville, TN; Division of Allergy, Pulmonary, and Critical Care Medicine, Vanderbilt University Medical Center, Nashville, TN; Division of Infectious Diseases, Vanderbilt University Medical Center, Nashville, TN; Division of Pulmonary and Critical Care Medicine, Department of Medicine, University of Maryland School of Medicine, Baltimore MD; Department of Medicine, the Department of Anesthesiology, Vanderbilt University Medical Center, Nashville, TN; Department of Biomedical Informatics, Vanderbilt University Medical Center, Nashville, TN; Department of Medicine, the Department of Anesthesiology, Vanderbilt University Medical Center, Nashville, TN; Department of Biomedical Informatics, Vanderbilt University Medical Center, Nashville, TN; Vanderbilt Vaccine Research Program, Vanderbilt University Medical Center, Nashville, TN; Division of Pediatric Infectious Diseases, Vanderbilt University Medical Center, Nashville, TN; Vanderbilt Vaccine Research Program, Vanderbilt University Medical Center, Nashville, TN; Division of Pediatric Infectious Diseases, Vanderbilt University Medical Center, Nashville, TN; Vanderbilt Vaccine Research Program, Vanderbilt University Medical Center, Nashville, TN; Division of Pediatric Infectious Diseases, Vanderbilt University Medical Center, Nashville, TN; Department of Pediatrics, the Department of Biostatistics, Vanderbilt University Medical Center, Nashville, TN; Department of Pediatrics, the Department of Biostatistics, Vanderbilt University Medical Center, Nashville, TN; Division of Allergy, Pulmonary, and Critical Care Medicine, Vanderbilt University Medical Center, Nashville, TN; Vanderbilt Institute for Clinical and Translational Research,Vanderbilt University Medical Center, Nashville, TN

**Keywords:** infection control, methicillin-resistant *Staphylococcus aureus*, patient isolation, pragmatic clinical trial, vancomycin-resistant *Enterococci*

## Abstract

**BACKGROUND:**

Contact precautions are used commonly to prevent nosocomial transmission of methicillin-resistant *Staphylococcus aureus* (MRSA) and vancomycin-resistant *Enterococci* (VRE). Whether targeted contact precautions are as effective at preventing nosocomial infection as contact precautions for all patients with MRSA and VRE is uncertain.

**RESEARCH QUESTION:**

Compared with standard contact precautions, are targeted contact precautions able to reduce the number of days patients spend in contact precautions and are they as effective at preventing hospital-acquired infection with MRSA and VRE?

**STUDY DESIGN AND METHODS:**

A pragmatic, single-center, single-cluster, crossover pilot and feasibility trial in which all adults admitted to a single medical ICU alternated every 2 months between targeted and standard contact precautions for MRSA and VRE for a total of 8 months. In targeted periods, patients received contact precautions only for highly transmissible MRSA or VRE infections, including open wounds, uncontrolled diarrhea, or pneumonia. In standard periods, patients received contact precautions for any MRSA or VRE infection or colonization. The primary pilot outcome was hospital-acquired infection with MRSA or VRE. The primary feasibility outcome was the number of patients per day removed from contact precautions in the targeted group.

**RESULTS:**

The trial enrolled 2,230 adults. During targeted months, 0.56 additional patients/d remained without contact precautions. Thirteen hospital-acquired infections with MRSA or VRE occurred during targeted months compared with 13 infections during standard months (*P* = .95). MRSA acquisition was higher in the targeted group (6.5% vs 3%; *P* = .03).

**INTERPRETATION:**

Despite a slight reduction in patient-days spent with contact precautions during the targeted contact precautions periods, no statistically significant difference was found in hospital-acquired MRSA or VRE infections when comparing targeted with standard contact precautions in this pilot trial. Larger, multicenter trials should examine the benefits and risks of these interventions.

**CLINICAL TRIAL REGISTRATION:**

ClinicalTrials.gov; No.: NCT03268122; URL: www.clinicaltrials.gov

Methicillin-resistant *Staphylococcus aureus* (MRSA) and vancomycin-resistant *Enterococci* (VRE) are among the most common nosocomial pathogens.^1^ These organisms pose a particularly serious problem in ICUs because of the critically ill population located therein. Hospitals have used numerous strategies to prevent these organisms from spreading between patients, including contact precautions, which includes the wearing of disposable gowns and gloves in patient rooms. However, contact precautions have been shown to decrease the number of times providers enter patient rooms by up to 50%.^2,3^ Contact precautions also have been associated with a higher incidence of falls and pressure injuries in some,^4^ but not all,^5^ studies. Furthermore, despite the widespread adoption of contact precautions for MRSA and VRE, no randomized trial has shown a clinically significant benefit for using contact precautions for all MRSA and VRE infections and colonization in hospitalized patients. Given the conflicting evidence, some hospitals have limited or discontinued contact precautions for these organisms.^6–14^

The Comparison of Standard and Targeted Isolation for Preventing Nosocomial Transmission of MRSA and VRE trial was designed to compare the effectiveness of targeted contact precautions vs standard contact precautions in critically ill adults for preventing hospital-acquired MRSA or VRE infections and to determine the feasibility of performing a larger, multicenter trial.

## Study Design and Methods

We performed a pragmatic, single-center, single-cluster, crossover pilot trial in the medical ICU (MICU) at a tertiary care academic hospital between September 1, 2017, and April 30, 2018, to compare the effectiveness and safety of targeted contact precautions vs standard contact precautions regarding the incidence of hospital-acquired infection with MRSA or VRE and the degree to which targeted contact precautions reduce the number of patients with contact precautions. The trial was approved by the Vanderbilt University institutional review board (Identifier: 161137) with a waiver of informed consent and was registered online before beginning enrollment.

### Patient Population and Study ICU

All adults admitted to the participating MICU during the study period were enrolled in the trial. Patients discharged from the hospital and readmitted to the MICU on a subsequent hospitalization were re-enrolled in the trial. The study ICU contains 35 single-occupancy beds at a tertiary care academic medical center.

### Randomization and Masking

Each intervention period consisted of 2 consecutive calendar months during which the ICU was assigned to use either a targeted contact precautions strategy or a standard contact precautions strategy for all patients. The duration of each intervention period was chosen to allow sufficient time for staff to adjust to the change in contact precautions strategy. The study consisted of a total of 4 consecutive intervention periods for a total study duration of 8 calendar months. The contact precautions strategy during the initial study period was selected randomly using simple, computerized randomization. Subsequent intervention periods alternated sequentially between the 2 strategies to ensure the maximum number of crossovers between treatment groups during the study (e-Fig 1). Given the nature of the intervention, masking of patients and providers was not feasible.

### Treatments

Patients admitted to the MICU during targeted contact precautions periods were placed in contact precautions only if they demonstrated a highly transmissible infection with MRSA or VRE. A highly transmissible infection was defined as an infection associated with uncontained bodily secretions or excretions, such as open wounds or uncontrolled diarrhea, or an infection capable of causing aerosolization, such as pneumonia.^15^ Patients with contained infections with MRSA or VRE, colonization, or a history of MRSA or VRE did not receive contact precautions. Patients with active but not highly transmissible infections with MRSA or VRE such as bacteremia, urinary tract infections, or meningitis did not receive contact precautions. Contact precautions were removed for patients, according to the clinical team’s determination, who were no longer experiencing an active, highly transmissible infection with MRSA or VRE at that time, if contact precautions were not required for another pathogen.

Patients admitted to the MICU during standard contact precautions periods followed usual hospital protocol. They received contact precautions if they were experiencing active infection with MRSA or VRE or had a history of infection or colonization with either organism within the previous 3 months. No screening for MRSA or VRE was performed during the study period except for study purposes as described herein, but patients with MRSA or VRE colonization detected at other facilities before transfer received contact precautions. No decolonization for MRSA or VRE was performed in the ICU during the study. Discontinuation of contact precautions for both infection and colonization required at least 2 negative results from nasal MRSA swabs or 3 negative results from rectal VRE swabs performed at least 1 week apart, as well as negative culture findings from the site of infection or colonization, if obtainable without excess risk to the patient.

The intervention began immediately on a patient’s physical arrival to the MICU, which was considered the time of study enrollment. The intervention ended on physical transfer out of the MICU or discharge, including death, directly from the MICU. For patients temporarily leaving the ICU for a procedure or diagnostic study, their MRSA and VRE status and current contact precautions strategy were communicated to the receiving unit, which could opt to institute contact precautions for the patient temporarily if desired. Patients in the ICU during a transition between the standard and targeted contact precautions strategies were immediately changed over to the new strategy. Other indications for contact precautions, including patients with *Clostridioides difficile* colitis, multidrug-resistant gram-negative infections, and certain viral infections, were not affected by this trial. Staff members were allowed to wear personal protective equipment (PPE) in the event they were uncomfortable entering a patient’s room without a gown or gloves, according to usual hospital protocol. All other practices related to contact precautions, hand hygiene, and room cleaning remained unchanged during the study period, with the exception that the disinfectant used to clean patient rooms changed partway through the study. This change occurred on January 1, 2018, such that the old and new disinfectants each were used for one-half of the trial’s duration and during an equal number of targeted and standard contact precautions periods.

### Data Collection

Unless otherwise specified, data were collected as part of routine clinical care and were extracted from preexisting hospital databases. Demographics and outcomes data were extracted from the electronic medical record. Enactment of contact precautions for patients and the reasons for contact precautions were obtained from a clinical database maintained by the hospital infection prevention team. These data were compared with the contact precautions strategy in place each day to determine the appropriate enactment of contact precautions for patients. The infection prevention team also provided data on staff compliance with hand hygiene and contact precautions as posted on the patient’s door obtained through a preexisting system of covert observation of hospital staff. Adverse events were collected systematically as part of routine quality improvement efforts. Medication errors were reported through a standard, anonymous, system-wide safety alert system. Preexisting data regarding the delivery of gowns, gloves, and disposable stethoscopes were used to estimate the monthly cost of contact precautions in the MICU by multiplying the monthly number of gowns, gloves, and disposable stethoscopes delivered to the MICU by the unit cost at the time of purchase by the hospital. The following data on select secondary outcomes were collected monthly in aggregate and were not assigned to individual patients: isolation equipment delivered to the ICU, adverse events, and compliance with hand hygiene and contact precautions as posted on the patient’s door. To assess for changes in nasal colonization of MRSA, all patients admitted to the study ICU underwent nasal swab collection by nurses on ICU admission and discharge during the second half of the study (January-April 2018). Samples were collected in CultureSwabs with Liquid Amies media (Becton, Dickinson and Co.), cultured directly onto CHROMagar *S aureus* plates (Becton, Dickinson and Co.) and incubated overnight at 35 ^◦^C. Mauve colonies then were incubated overnight on tryptic soy agar with 5% sheep blood (Becton, Dickinson and Co.). Methicillin resistance was determined by oxacillin screen agar (Becton, Dickinson and Co.). The clinical team did not receive results from MRSA detection because it was not collected as part of hospital protocol.

### Outcomes

Initially, the primary pilot outcome was defined as occurrence of ICU-acquired MRSA or VRE infections, defined as clinical infection with MRSA or VRE occurring between 48 hours after study ICU admission and 48 hours after study ICU discharge, censored at 28 days after enrollment. However, because infections acquired in the ICU might not manifest or be diagnosed until after ICU discharge, before study completion or any data analysis, the primary pilot outcome was changed to occurrence of hospital-acquired infection with MRSA or VRE, defined as clinical infection with MRSA or VRE occurring between 48 hours after ICU admission and the time of hospital discharge. Data were censored at 30 days after enrollment, rather than 28 days, to be consistent with data provided by the infection prevention team. Infections were adjudicated by the hospital’s infection prevention team as part of preexisting quality improvement mechanisms using standard National Healthcare Safety Network definitions. The primary feasibility outcome was the number of patients per day in the targeted isolation group who had nonhighly transmissible MRSA or VRE infections and for whom contact precautions were not enacted. Secondary pilot outcomes included ICU-acquired MRSA or VRE infections, also adjudicated by the infection prevention team. Infections were attributed to the study ICU if they occurred between 48 hours after study ICU admission and 48 hours after study ICU discharge or transfer and were censored at 30 days after enrollment. Secondary outcomes also included the occurrence of individual infections included in the primary outcome, in-hospital mortality, hospital length of stay after enrollment, central line-associated bloodstream infections, catheter-associated urinary tract infections, *C difficile* infections, and estimated cost of isolation equipment. Secondary outcomes also included MRSA acquisition, defined as patients with both ICU admission and discharge swabs obtained who showed negative results for MRSA colonization on admission but positive results on ICU discharge. In these patients, incidence density of acquisition also was described per 1,000 patient-days at risk of the outcome. Secondary feasibility outcomes included the number of patients for whom contact precautions were enacted, number of patients meeting criteria for contact precautions without contact precautions ordered, and compliance with hand hygiene and posted contact precautions, collected to help determine feasibility of a larger trial. Safety outcomes included falls, pressure injuries, and medication administration errors.

### Sample Size

Because this pilot study was designed primarily to unfold during a dedicated period (8 months), no formal sample size calculation was performed. The study duration was based on logistics of data collection and to limit any concerns over potential increase in infection with MRSA, VRE, or both from a more widespread, longer-lasting intervention. Over the 4 2-month crossovers (ie, the 8 months of total study duration), we anticipated that 2,267 patients would be admitted to the MICU and enrolled in the study based on ICU admission data from the previous year.

### Statistical Analysis

Categorical variables are reported as frequencies and proportions. Continuous variables are reported as mean (SD) and median (interquartile range), as appropriate. The Pearson χ^2^ test and the Fisher exact test were used for the categorical comparisons of initial, unadjusted relationships. For the primary analysis, a univariable logistic regression model was used to investigate the association between group assignment (ie, targeted vs standard) and hospital-acquired infection with MRSA or VRE. Generalized estimating equation models then were fitted to account for the cluster level study design. Model results were summarized with ORs and the corresponding 95% CIs. All patients in the primary analysis with any exposure to the intervention arm (ie, the targeted contact precautions arm) were analyzed in the targeted contact precautions group, whereas the standard contact precautions group comprised only patients never exposed to the intervention arm (e-Fig 1). A sensitivity analysis was performed including only patients who did not cross over between standard and targeted contact precautions arms. Prespecified secondary outcomes were compared by treatment group in a similar manner as the primary analysis. All statistical analyses were performed using R version 4.2.0 statistical software (R Foundation for Statistical Computing) with statistical significance set at a 2-sided *P* value of < .05.

## Results

### Study Population

During the study period, 1,923 unique patients were admitted to the MICU for a total of 2,230 admissions (Fig 1). A total of 1,129 patient admissions were exposed at any time during the ICU stay to targeted contact precautions, whereas 1,101 patients were exposed to only the standard contact precautions strategy. No patients admitted to the study ICU during the study period were excluded. The mean ICU census was 32.7 patients/d. The median patient age was 59 years, and 48% of patients were female (Table 1). Patient characteristics were well balanced between groups.

### Primary Feasibility Outcome

An average of 7.2 patients/d received contact precautions during targeted contact precaution months, and 7.6 patients/d received contact precautions during standard months (e-Table 1). During targeted months, 0.72 patients/d (2.2%) demonstrated nonhighly transmissible MRSA or VRE infection or colonization and were eligible for removal of contact precautions; for whom 0.56 patients/d (1.7%) contact precautions were discontinued. During standard months, 1.17 patients/d (3.6%) demonstrated nonhighly transmissible MRSA or VRE infection or colonization as the sole reason for contact precautions and would have been eligible for removal of precautions under a targeted contact precautions strategy. A total of 7 patients meeting criteria for contact precautions did not have them ordered, 3 patients during targeted months and 4 patients during standard months. Other reasons for initiation of contact precautions, including *C difficile*, are described in e-Table 1.

### Primary Pilot Outcome

A total of 26 hospital-acquired MRSA or VRE infections in 25 patients were recorded during the study period (Table 2). Thirteen infections occurred in the targeted contact precautions group and 13 infections occurred in the standard contact precautions group. The logistic regression model yielded no statistically significant associations between the intervention group and hospital-acquired infection with MRSA or VRE (OR, 0.97; 95% CI, 0.45–2.11; *P* = .95). The generalized estimating equation model showed similar results (e-Table 2). Among the infections, 12 infections were caused by MRSA and 14 infections were caused by VRE. One patient in the standard contact precautions arm demonstrated an infection with both MRSA and VRE on the same date. Seven infected patients died in the hospital, 4 patients in the targeted contact precautions group and 3 patients in the standard contact precautions group.

### Sensitivity Analysis

The sensitivity analysis of patients who were exposed only to either the standard or the targeted contact precautions strategies during an ICU stay demonstrated similar results to the primary analysis. When analyzed in this manner, 8 hospital-acquired MRSA or VRE infections occurred in 1,001 patients in the targeted contact precautions group and 13 infections occurred in 1,101 patients in the standard group (OR, 0.67; 95% CI, 0.28–1.63; *P* = .38).

### Secondary Outcomes

A total of 17 ICU-acquired MRSA or VRE infections occurred during the study, 11 infections in the targeted contact precautions group and 6 infections in the standard precautions group. The logistic regression model indicated no statistically significant associations between intervention group and ICU-acquired infection with MRSA or VRE (OR, 1.54; 95% CI, 0.59–4.65; *P* = .37). Hospital mortality was 22% in the targeted contact precautions group and 30% in the standard precautions group (OR, 0.65; 95% CI, 0.54–0.79; *P* < .001). Hospital length of stay after study enrollment was similar between the targeted and standard precautions groups (OR, 1.13; 95% CI, 0.98–1.32; *P* = .10). The generalized estimating equation models showed similar results (e-Table 2).

During the second half of the study, ICU admission and discharge MRSA swabs were collected in 93% (1,017 patients) of the 1,098 patients on admission and in 70% (773 patients) of those 1,098 patients on discharge from the ICU. A total of 14% of patients (142 patients) showed colonization with MRSA on admission to the ICU, and 15% of patients (113 patients) showed colonized on ICU discharge. A total of 6.5% of patients with both admission and discharge swabs available (18 of 277 patients) acquired MRSA in the ICU during the targeted contact precautions months, whereas 3.0% of patients (10 of 333 patients) did so during the standard contact precautions months (*P* = .03, χ^2^ test). The incidence density of MRSA acquisition was 17.1 per 1,000 patient-days during the targeted contact precautions months and 8.8 per 1,000 patient-days during the standard contact precautions months.

During the study period, among observed interactions in the MICU, 95% of staff entering patient rooms performed hand hygiene properly, 97% during targeted contact precautions months and 93% during standard months (*P* = .23, Fisher exact test) (e-Table 3). In the 12 months before the study, hand hygiene compliance was 98% in the MICU. Compliance with posted contact precautions also was high in both treatment arms, although the number of observations was limited (e-Table 4).

### Adverse Events

The incidence of pressure injuries and falls was infrequent and similar between the 2 groups (Table 3). Medication errors occurred 13 times in the targeted contact precautions months compared with 22 times in the standard contact precautions months. Other adverse events are listed in Table 3.

### Cost

A total of $28,155 worth of gowns, gloves, and disposable stethoscopes were delivered to the study ICU during targeted contact precautions months, as compared with $28,808 worth of gowns, gloves, and disposable stethoscopes during standard contact precautions months.

## Discussion

This pilot trial of contact precautions strategies for patients with MRSA and VRE showed similar rates of hospital-acquired infections and adverse events when a targeted contact precautions strategy was compared with a standard contact precautions strategy. Previous studies supporting contact precautions for MRSA and VRE have been primarily observational.^16–29^ Many initial studies were performed in the setting of MRSA outbreaks, as opposed to endemic MRSA.^16,18,22,23,26^ Many of these studies also compared a bundle of interventions, making it difficult to determine which intervention, if any, was responsible for improvements in MRSA or VRE infection rates or both.^17–25,27–29^ Furthermore, many older studies did not measure staff compliance with hand hygiene, but those that did found it to be at or < 50%.^19,24^ Hand hygiene has been shown to prevent pathogen transmission,^30^ and it is possible that contact precautions serve as a second line of defense preventing nosocomial transmission of organisms remaining on the hands of providers who do not perform adequate hand hygiene.

To our knowledge, this is the first trial comparing standard contact precautions with targeted contact precautions for MRSA and VRE. Of previous studies examining contact precautions in MRSA and VRE, 1 cluster-randomized trial compared universal gloving combined with MRSA and VRE screening (and placement into contact precautions for those with positive screening results) with standard contact precautions.^31^ The trial demonstrated no difference in MRSA and VRE acquisition.^31^ Another randomized trial compared universal MRSA decolonization and contact precautions for those patients with MRSA or VRE infections with 2 arms of universal MRSA screening with contact precautions for those with positive screening results, 1 arm additionally requiring MRSA decolonization for those with positive screening results.^32^ This study showed a lower rate of ICU-attributable MRSA infections in the universal decolonization arm. Another randomized trial comparing universal contact precautions with contact precautions only for patients with MRSA and VRE infections^33^ showed no difference in the primary outcome of either MRSA or VRE acquisition, but did show a statistically significant decrease in a secondary outcome of MRSA acquisition. Our study is consistent with these trials, showing no difference in hospital-acquired MRSA or VRE infections. Furthermore, the only trial that demonstrated a benefit of contact precautions reported much better hand hygiene compliance in the universal contact precautions group (78% vs 60%; *P* = .02),^33^ suggesting that improved hand hygiene may be responsible for the observed benefit. Because hospital staff during our study period showed hand hygiene compliance rates well above 90%, our results are more likely to be driven by the contact precautions themselves. Unlike other studies, we found a statistically significant difference in the secondary outcome of hospital mortality favoring targeted contact precautions. However, given that we did not find a statistically significant difference in hospital-acquired infections with MRSA or VRE, the mechanism for any mortality benefit is unclear. This finding would need to be confirmed in future studies. MRSA acquisition was higher in patients treated in the ICU during the targeted contact precautions periods, although the clinical significance of this remains uncertain given the similar rates of acquired infections and lower mortality in the targeted contact precautions periods combined with lack of standardized decolonization of patients in the study ICU.

The emergence of the COVID-19 pandemic gave additional impetus to measures designed to conserve PPE. Limiting the use of contact precautions for MRSA or VRE may assist these efforts. Our feasibility outcome demonstrated a modest reduction of 0.56 patients/d for whom contact precautions were enacted during targeted contact precaution periods. Our limited evaluation of PPE supply cost did not find a large cost savings in the targeted contact precautions strategy. However, other studies have estimated the cost per patient of daily contact precautions to be between $31 and $158.90, depending on the inclusion of indirect costs such as provider time, which was not evaluated in our study.^34,35^ In these studies, estimated daily gown and glove use per patient requiring contact precautions ranged from 7 to 31 items and from 32 to 82 items, respectively, demonstrating a potential for significant conservation of PPE and cost savings from the adoption of a more targeted contact precautions strategy for MRSA and VRE.

### Strengths and Limitations

Strengths of this study include its broad eligibility criteria. Another strength is the application of the study intervention throughout the entire ICU stay, maximizing separation between treatment arms. Furthermore, the high rate of hand hygiene among staff allows for a more focused evaluation of contact precautions themselves.

Limitations of this study include its small size and single-center pilot nature. The single ICU and limited duration left the study at risk of seasonal variation, which could have resulted in an imbalance of patients requiring contact precautions. However, the similar proportions of patients requiring contact precautions for reasons other than MRSA or VRE in both arms argue against significant seasonal imbalance. The performance of the trial only in a medical ICU limits its generalizability to other patient populations. Another potential limitation is the collection of MRSA nasal swabs during only the second half of the study and lack of collection of VRE rectal swabs to evaluate for colonization. The clinical implication of the increased MRSA acquisition are unclear, given the lack of difference in the primary outcome of hospital-acquired infections and the lack of routine screening at the study hospital, meaning that colonization data did not affect empiric antimicrobial selection. Furthermore, the secondary outcome of ICU-acquired infections was similar to the primary outcome, strengthening our confidence in the findings. The pragmatic nature of the trial prevented collection of some data, including patients’ prior history of MRSA or VRE and severity of illness scores. We did not collect data on other core infection prevention measures such as room cleaning. We also did not evaluate staff for acquisition of MRSA or VRE. We did not obtain genetic data on relatedness of MRSA or VRE isolated during the study, and thus were unable to prove whether infections were caused by an organism acquired during the ICU stay. Finally, because staff were permitted to don gowns and gloves for any patient encounter, some staff may have worn gowns and gloves for patients with nonhighly transmissible MRSA or VRE during targeted precautions months, diluting the potential effect of the intervention.

### Interpretation

In this pilot and feasibility trial, targeted contact precautions were implemented with high compliance. This slightly reduced the number of days spent in contact isolation, but did not seem to affect the occurrence of hospital-acquired infections with MRSA or VRE compared with standard contact precautions, suggesting feasibility of a larger trial. Although rare, nasal acquisition of MRSA was higher in the targeted precautions group. Larger, multicenter trials should examine the benefits and risks of targeted vs standard contact precautions further for critically ill adults.

## Supplementary Material

1

**Additional information:** The e-Figure and e-Tables are available online under “Supplementary Data.”

## Figures and Tables

**Figure 1 –:**
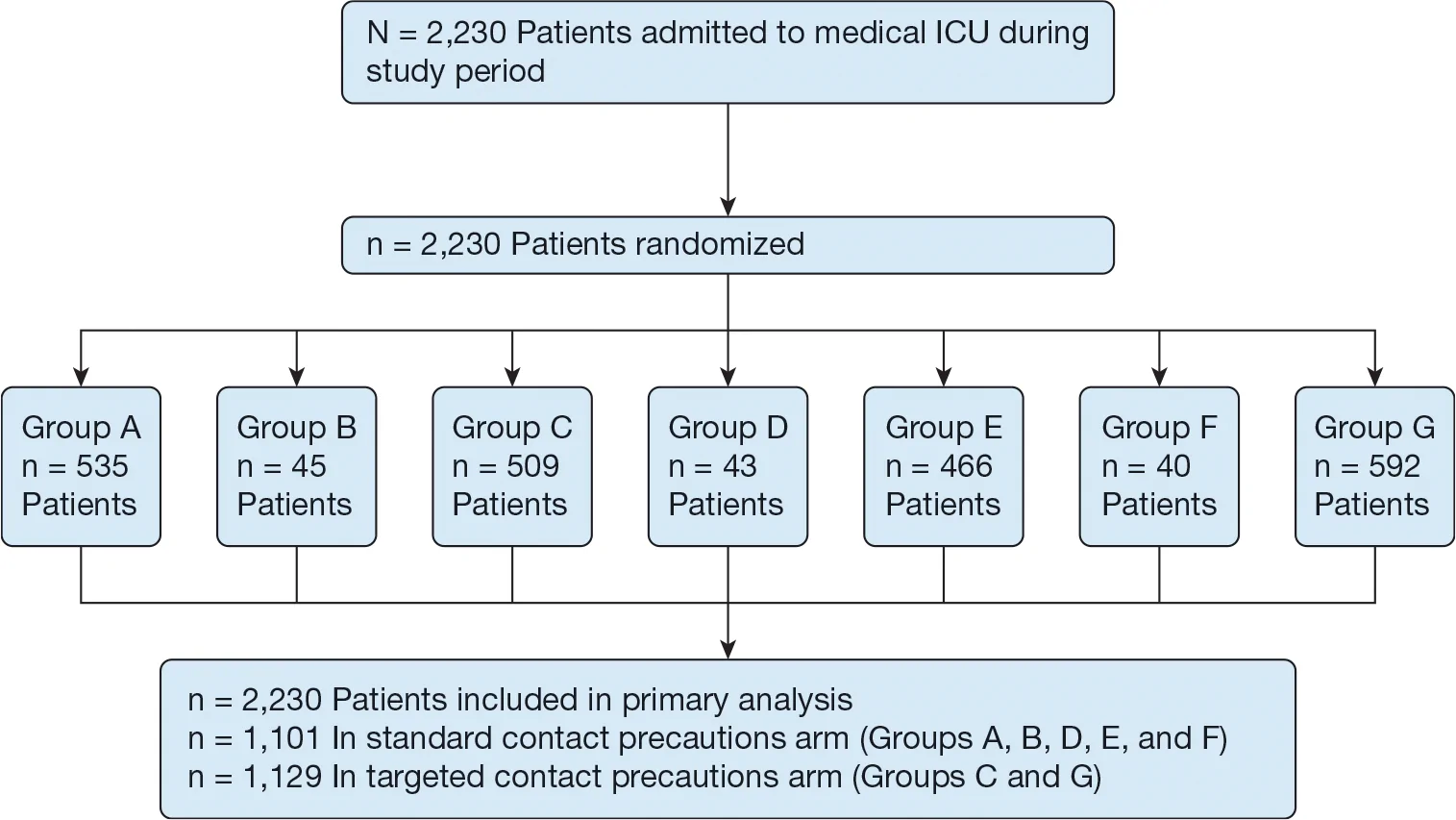
Consolidated Standards of Reporting Trials diagram. Patients were treated with the contact precautions strategy in place during the entire ICU admission. For patients whose ICU stay overlapped 2 study periods, the contact precautions strategy changed on the same day as the rest of the ICU. Patients in groups A and E were treated exclusively with the targeted contact precautions strategy. The ICU stay of patients in groups B, D, and F overlapped 2 study periods, and thus these patients experienced both targeted and standard contact precautions. For the primary analysis, patients were analyzed as part of the targeted contact precautions group if they had any exposure to targeted contact precautions (groups A, B, D, E, and F). Only patients treated exclusively with the standard contact precautions strategy (groups C and G) were included in the standard contact precautions group for the primary analysis. See e-Figure 1 for a graphic representation of the study groups.

**TABLE 1 ]:** Participant Demographics

Variable	Standard Contact Precautions (n = 1,101)	Targeted Contact Precautions (n = 1,129)^a^

Age, y	60 (45–69)	58 (44–68)
Female sex	525 (48)	545 (48)
Race		
White	799 (73)	868 (77)
Black	233 (21)	188 (17)
Asian	15 (1)	15 (1)
Mixed	14 (1)	12 (1)
Other^b^	10 (1)	6 (1)
Unknown	30 (3)	40 (4)
Ethnicity (n = 1,640)		
Hispanic	20 (2)	20 (3)
Not Hispanic	971 (93)	559 (93)
Unknown	51 (5)	19 (5)
Source of hospitalization		
Emergency department	661 (60)	673 (60)
Hospital ward	140 (13)	197 (17)
ICU	41 (4)	58 (5)
Operating room	23 (2)	14 (1)
Home or outside facility	236 (21)	187 (17)
Length of hospital stay before ICU admission, d	0 (0–1)	0 (0–1)
Receiving invasive mechanical ventilation on ICU admission	283 (26)	400 (35)
WBC count on ICU admission (n = 1,967), ×1,000/mL	11.4 (7.5–16.6)	11.4 (7.7–16.9)
Creatinine on ICU admission (n = 2,046), mg/dL	1.22 (0.81–2.30)	1.12 (0.76–2.16)

Data are presented as No. (%) or median (interquartile range).

a Patients are included in the targeted contact precautions arm if they were exposed at any time during the ICU stay to the targeted contact precautions strategy.

b Includes a mix of < 5 patients listed as Native American/American Indian and race listed only as “other” in the self-reported data.

**TABLE 2 ]:** Patients With Hospital-Acquired Infections of MRSA, VRE, or Both

Patient No.	Group^a^	Age, y	Sex	Organism	Culture Type	ICU Onset^b^	Duration Between Enrollment and Infection, d^c^	Hospital Mortality

1	Targeted	62	Male	VRE	Respiratory	Yes	5	Yes
2	Standard	51	Male	VRE	Urine	No	6	No
3	Standard	68	Female	MRSA and VRE	Tissue or surgical	No	5^d^	No
4	Standard	41	Female	MRSA	Respiratory	Yes	11	No
5	Targeted	28	Male	MRSA	Wound	Yes	3	No
6	Standard	63	Male	VRE	Body fluid	No	22	Yes
7	Standard	43	Female	MRSA	Blood	No	12	No
8	Targeted	44	Female	VRE	Blood	Yes	5	No
9	Targeted	63	Female	VRE	Body fluid	No	9	No
10	Targeted	71	Male	VRE	Tissue or surgical	Yes	21	No
11	Targeted	44	Female	VRE	Body fluid	No	17	No
12	Standard	42	Male	MRSA	Respiratory	Yes	3	No
13	Standard	79	Male	VRE	Blood	Yes	3	No
14	Targeted	57	Male	VRE	Blood	Yes	15	Yes
15	Targeted	62	Female	VRE	Blood	Yes	21	Yes
16	Standard	75	Male	MRSA	Blood	No	9	Yes
17	Targeted	34	Female	MRSA	BAL fluid	Yes	8	No
18	Targeted	43	Female	MRSA	Blood	Yes	4	No
19	Targeted	56	Female	VRE	Body fluid	Yes	3	No
20	Standard	36	Male	MRSA	Abscess	Yes	4	No
21	Targeted	56	Female	VRE	Urine	Yes	3	Yes
22	Targeted	59	Male	MRSA	BAL fluid	Yes	3	No
23	Standard	24	Male	MRSA	BAL fluid	Yes	2	Yes
24	Standard	57	Female	VRE	Urine	No	15	No
25	Standard	70	Male	MRSA	Respiratory	Yes	6	No

BAL = bronchoalveolar lavage; MRSA = methicillin-resistant *Staphylococcus aureus*; VRE = vancomycin-resistant *Enterococci*.

a Patients are listed in the group in which they were analyzed with respect to the primary outcome. Patients are included in the targeted contact precautions arm if they had any exposure to the targeted contact precautions group. Patients 11 and 14 were admitted during the standard contact precautions arm, but were exposed to targeted precautions while in the ICU, and so are included in the targeted precautions arm. Patients 1, 21, and 22 were admitted during the targeted precautions arm, but also were exposed to standard contact precautions while in the ICU. They are also included in the targeted precautions arm.

b Infections are defined as having an ICU onset when cultures drawn between 48 hours after ICU admission and 48 hours after ICU discharge showed positive findings for MRSA, VRE, or both.

c Enrollment is defined as time of admission to the study ICU.

d The culture samples that grew both MRSA and VRE were drawn on the same date.

**TABLE 3 ]:** ICU-Attributable Adverse Events

Adverse Event	Standard Contact Precautions	Targeted Contact Precautions	Total

Falls	6	4	10
Falls with harm	1	0	1
Pressure injuries	6	7	13
Medication errors	22	13	35
CLABSI	0	0	0
CAUTI	1	0	1
*C difficile* colitis	4	5	9

Data are presented as No. All ICU-attributable adverse events are collected as part of preexisting quality improvement mechanisms. Events are categorized by the contact precautions strategy in place when the event occurred. CAUTI = catheter-associated urinary tract infection; CLABSI = central line-associated bloodstream infection.

## Take-Home Points

### Research Question:

Are targeted contact precautions as effective as standard contact precautions at preventing hospital-acquired methicillin-resistant *Staphylococcus aureus* (MRSA) and vancomycin-resistant *Enterococci* (VRE) infections in critically ill adults?

### Results:

This pilot trial showed no major increase in hospital-acquired infections with MRSA or VRE in critically ill adults treated with a targeted contact precautions strategy, with 13 infections in the targeted contact precautions group and 13 infections in the standard contact precautions group.

### Interpretation:

This pilot trial demonstrated the feasibility and safety of performing a larger, multicenter randomized trial of contact precautions in critically ill patients to prevent nosocomial infections with MRSA and VRE.

## ABBREVIATIONS:

MICU: medical ICU
MRSA: methicillin-resistant *Staphylococcus aureus*
OR: operating room
PPE: personal protective equipment
VRE: vancomycin-resistant *Enterococci*
