# Supplementary material for: Targeted vs Standard Contact Precautions and Transmission of Methicillin-Resistant Staphylococcus aureus and Vancomycin-Resistant Enterococci in Critically Ill Adults: A Pilot Clinical Trial
Source: CHEST Crit Care. Author manuscript; Available in PMC 2026 Nov 11. (PMC13750674; doi:10.1016/j.chstcc.2026.100282)
Supplement: Supplementary file 1 [file NIHMS2210621-supplement-1.docx]

This Electronic Data Supplement contains the following items:

Supplemental Table 1: Patients under Contact Precautions

Supplemental Table 2: Primary and Secondary Outcomes - GEE Models

Supplemental Table 3: Hand Hygiene Compliance

Supplemental Table 4: Contact Precautions Compliance

Supplemental Figure 1: Study Schedule

**eTable 1: Patients under Contact Precautions**

|  | **Standard** | **Targeted** |
| --- | --- | --- |
| Patients under Contact Precautions | 657 | 674 |
| Mean Daily Patients under Contact Precautions | 7.6 | 7.2 |
| Reasons for Contact Precautions: |  |  |
| Non-Highly Transmissible MRSA or VRE | 159 (24.2) | 65 (9.6) |
| MRSA | 138 (21.0) | 55 (8.2) |
| VRE | 26 (4.0) | 12 (1.8) |
| Non-Highly Transmissible MRSA or VRE Sole Reason for Contact Precautions | 100 (15.2) | 2 (0.3) |
| MRSA | 84 (12.8) | 1 (0.1) |
| VRE | 21 (32.0) | 1 (0.1) |
| Highly Transmissible MRSA or VRE | 154 (23.4) | 159 (23.6) |
| MRSA | 153 (23.3) | 153 (22.7) |
| VRE | 4 (0.6) | 6 (0.9) |
| *Clostridioides difficile* | 82 (12.5) | 103 (15.3) |
| Multi-Drug Resistant Gram Negative Bacteria | 221 (33.6) | 184 (27.3) |
| Cystic Fibrosis | 25 (3.8) | 39 (5.8) |
| Respiratory Viruses | 136 (19.2) | 236 (35.0) |
| Herpes Virus | 16 (2.4) | 7 (1.0) |
| Communicable Pests^a^ | 10 (1.5) | 5 (0.7) |
| Open Draining Wounds | 4 (0.6) | 0 (0) |
| Meningitis Rule-out | 1 (0.2) | 0 (0) |
| Other^b^ | 0 (0) | 2 (0.3) |
| Unknown | 0 (0) | 3 (0.4) |
| Values are presented as number (percent). Direct observations confirming presence of and reason for contact precautions were performed on 179/242 study days (74%), 93 days during the targeted contact precautions arm and 86 days during the standard contact precautions arm. Patients could have more than one reason for contact precautions; percentages may not add to 100%.  ^a^Communicable pests include lice, scabies, and bedbugs.  ^b^Other stated reasons for contact isolation include neutropenia and cellulitis.  MRSA = methicillin-resistant *Staphylococcus aureus*; VRE = vancomycin-resistant *Enterococci* | | |

**eTable 2: Primary and Secondary Outcomes - GEE Models**

|  | **OR** | **95% C.I.** | **P-value** |
| --- | --- | --- | --- |
| Hospital acquired infection with MRSA or VRE – Targeted vs Standard Contact Precautions | 0.97 | 0.45-2.11 | 0.96 |
| ICU acquired infection with MRSA or VRE – Targeted vs Standard Contact Precautions | 1.56 | 0.60-4.02 | 0.36 |
| Hospital Mortality – Targeted vs Standard Contact Precautions | 0.65 | 0.54-0.79 | <0.001 |
| GEE = generalized estimating equation; OR = odds ratio; C.I. = confidence interval; ICU = intensive care unit; MRSA = methicillin-resistant *Staphylococcus aureus*; VRE = vancomycin-resistant *Enterococci* | | | |

**eTable 3: Hand Hygiene Compliance**

| **Month** | **Arm** | **Observations** | **Compliant** |
| --- | --- | --- | --- |
| September 2017 | Targeted | 56 | 56 (100) |
| October 2017 | Targeted | 28 | 27 (96) |
| November 2017 | Standard | 34 | 32 (94) |
| December 2017 | Standard | 40 | 34 (85) |
| January 2018 | Targeted | 27 | 25 (93) |
| February 2018 | Targeted | 34 | 32 (94) |
| March 2018 | Standard | 36 | 35 (97) |
| April 2018 | Standard | 28 | 28 (100) |
| Total Targeted | Targeted | 145 | 140 (97) |
| Total Standard | Standard | 138 | 129 (93) |
| Compliance values are presented as number (percentage).  Intervention groups are based on the contact precautions strategy in place at the time of the compliance observation.  Compliance was defined as use of hand hygiene both prior to entering room and upon room departure, as noted by a clandestine observer for quality improvement purposes | | | |

**eTable 4: Contact Precautions Compliance**

| **Month** | **Arm** | **Observations** | **Compliant** |
| --- | --- | --- | --- |
| September 2017 | Targeted | 5 | 5 (100) |
| October 2017 | Targeted | 3 | 2 (67) |
| November 2017 | Standard | 3 | 3 (100) |
| December 2017 | Standard | 2 | 2 (100) |
| January 2018 | Targeted | 3 | 3 (100) |
| February 2018 | Targeted | 8 | 8 (100) |
| March 2018 | Standard | 7 | 7 (100) |
| April 2018 | Standard | 6 | 6 (100) |
| Total Targeted | Targeted | 19 | 18 (95) |
| Total Standard | Standard | 18 | 18 (100) |
| Compliance values are presented as number (percentage).  Intervention groups are based on the contact precautions strategy in place at the time of the compliance observation.  Compliance was defined as all of the following: hand hygiene prior to donning personal protective equipment (both gown and gloves), personal protective equipment worn prior to entering room, all personal protective equipment removed prior to leaving room, and hand hygiene perform after removing personal protective equipment, as noted by a clandestine observer for quality improvement purposes. | | | |

**eFigure 1: Study Schedule**

Patients were treated with the contact precautions strategy in place during their entire ICU admission. Patients whose ICU stay overlapped two study periods had their contact precautions strategy changed on the same day as the rest of the ICU. The black boxes (groups BDF) indicate participants whose ICU stay overlapped two study periods and thus experienced both targeted and standard contact precautions. For the primary analysis, patients were analyzed as part of the targeted contact precautions group if they had any exposure to targeted contact precautions (groups ABDEF). Only patients treated exclusively with the standard contact precautions strategy (groups C and G) were included in the standard contact precautions group for the primary analysis.
